# Analysis of space solar array arc images based on deep learning techniques

**DOI:** 10.1038/s41598-025-97579-y

**Published:** 2025-07-25

**Authors:** Afaf M. Abd El-Hameed, Ahmed S. Farahat, Khaled Y. Youssef, M. Elfarran, I. M. Selim

**Affiliations:** 1 National Research Institute of Astronomy and Geophysics (NRIAG), Helwan, Cairo 11421 Egypt; 2 Faculty of Navigation Science and Space Technology, Beni Suef University, Beni Suef, Egypt; 3 Faculty of Computer Science & Artificial Intelligence, University of Sadat City, Sadat City, Egypt

**Keywords:** Solar cells and array, Plasma arcs, Arc images, Image processing, Deep learning and convolutional neural networks, Python method, Maxim-DL technique, Analysis, Mathematics and computing, Physics

## Abstract

One of the critical challenges for solar arrays operating in space is the occurrence of discharging and arcing on the array surfaces exposed to plasma environments. Arcs can lead to severe damage to cell interconnectors due to the generated high peak currents and therefore significantly impact the performance and reliability of spacecraft systems. Solar arrays with highly negative biases are particularly prone to frequent arcs, which can escalate into sustained arcs, causing harm to spacecraft and satellite components. The presented study aims to investigate discharging and arc spectra on solar cell surfaces through the analysis of arc images. Leveraging advanced Deep Learning (DL) methodologies, including Convolutional Neural Networks (CNN) and Transfer Learning, a robust predictive model has been developed to analyze arc behavior and identify defective cells based on image data. Furthermore, algorithms and image processing tools, such as Python and Maxim-DL, are employed to examine variations in intensities and spatial variation in the arced region. The findings highlight the prevalence of intensive arcs, particularly on mid-cells and interconnectors, and provide insights into the dynamics of sustained arc events. The image analysis can advance the understanding of arc evolution, enabling improved mitigation strategies for solar array systems in space applications.

## Introduction

The interaction of satellites and spacecraft with the surrounding environment results in differential charging of their surfaces. There are two possible types of differential charging called “Normal Gradient” and “Inverse Gradient” conditions. The first type is characterized by the potential of the dielectric surface being negative with respect to the adjacent conductor. The second refers to the opposite distribution of electrostatic potentials. In both cases, a discharge or arc may occur if the electric field strength is high enough. Arcs manifest themselves as pulses of current with transient parameters (peak current, pulse width and shape) depending on the adjacent materials, level of charging, and dimensions of discharged surfaces^1–4^. In ground-based experiments of arcs on a solar array coupon, simulated solar array differential charging creates a strong electric field on the interconnector surface, and this surface serves as a cathode for the discharge. When a high-voltage solar array is exposed to surrounding plasma, discharging and arcs occur, particularly between adjacent cells and at conductor–dielectric junctions and interconnects of the array. Such discharge results in a reduction of electric power of the solar array and can cause significant damage and spacecraft anomalies^5–7^. The operation of a solar array with higher voltage can lead to parasitic current power losses in the electrical system. Arcs on solar array surfaces and the resulting damages have become a major concern due to failures of solar array strings on multiple spacecraft. Moreover, arcs can occur due to the presence of contaminants on the spacecraft surface leading to damage, degradation of spacecraft components^4,8–10^. Previously, experiments studied the behavior and locations of primary and sustained arc inception. The sustained arc gives excessive heat to the underlying insulation substrate. Thermal breakdown of the insulation substrate leads to a permanent short-circuit of solar array strings. Several satellites lost a part of solar array output power due to the sustained arc. In the laboratory, the microscopic analysis of the arced coupon surface revealed multiple traces of arcs and spotty contamination of the array coverglass^11–15^.

### Discharging and solar array arcs

Primary plasma discharging and arcs on space solar arrays are caused by high electric fields between cell interconnectors or substrates and the overlying solar cell coverglasses^16^. Typically, these arcs discharge the capacitance of nearby solar array coverglass surfaces^17^. The energies and currents of the primary arcs are usually not enough to degrade the cell performance of single-junction solar cells^18^. They can cause contamination issues on coverglass surfaces^19^. A previous study has indicated that discharges and arcs occur on the surfaces of high voltage solar arrays with a negative potential relative to the surrounding plasma.

The high potential my result in arcing discharges located on conductive surfaces (interconnectors or/and cell edges). Arcs may have detrimental effects on spacecraft operation such as power disruption, electromagnetic interference, and solar array output power degradation. Multi-junction solar cell types may experience more cell degradation due to contamination at the cell edges that can produce partial shorting of the cell^20^. The most probable site for an arc inception is a triple junction; metallic interconnect, cover glass and plasma^21–23^. Arcs on a triple junction can cause substantial damage to the solar array due to the discharge between adjacent cells with the highest potential difference. The mechanism of array failure is applicable to an array with a high enough operating voltage and potential difference between adjacent solar cells^21^. In the works of Snyder et al.^24^, the authors have confirmed that, when the voltages sufficient for sustained arcing are present, the primary arcs predominantly occur at the cell edges. Usually, the inter-cell voltages necessary for sustained arcing are supplied by the distributed voltages on the solar array coupon and strings. Once a sustained arc begins, its voltage and current are supplied by the solar array itself, and the arc may continue until it acts to break the circuit. Secondary arcing involves failures triggered by primary arcing that lead to sustained discharges and arc events due to charging gradients occurring on the triple junction cell. These processes result from plasma impact and high negatively biased voltage in simulated ground tests^24^. On the other hand, theoretical analysis and experimental data have demonstrated that water molecules absorbed on the side surface of dielectric materials (coverglass and adhesive) can play a decisive role in the process of arc inception^25–27^.

Vayner et al.^28^ investigated discharging and arcs that occurred on various materials immersed in plasma. The experiments were performed on different metal-dielectric junctions with a biased power supply providing negative voltage up to 1000 V. Their findings indicated that, with the negatively biased solar array, the arc spectra confirmed the ejection of metal atoms from the arc site resulting in contamination of sensitive spacecraft surfaces. The results confirmed that the most probable arc site occurred at a conductor–dielectric junction, specifically the junction site for solar array surfaces with negative potential. These findings can significantly contribute to the design of solar array interconnects and the understanding of plasma contamination processes caused by arcing^29^.

**Fig. 1:**
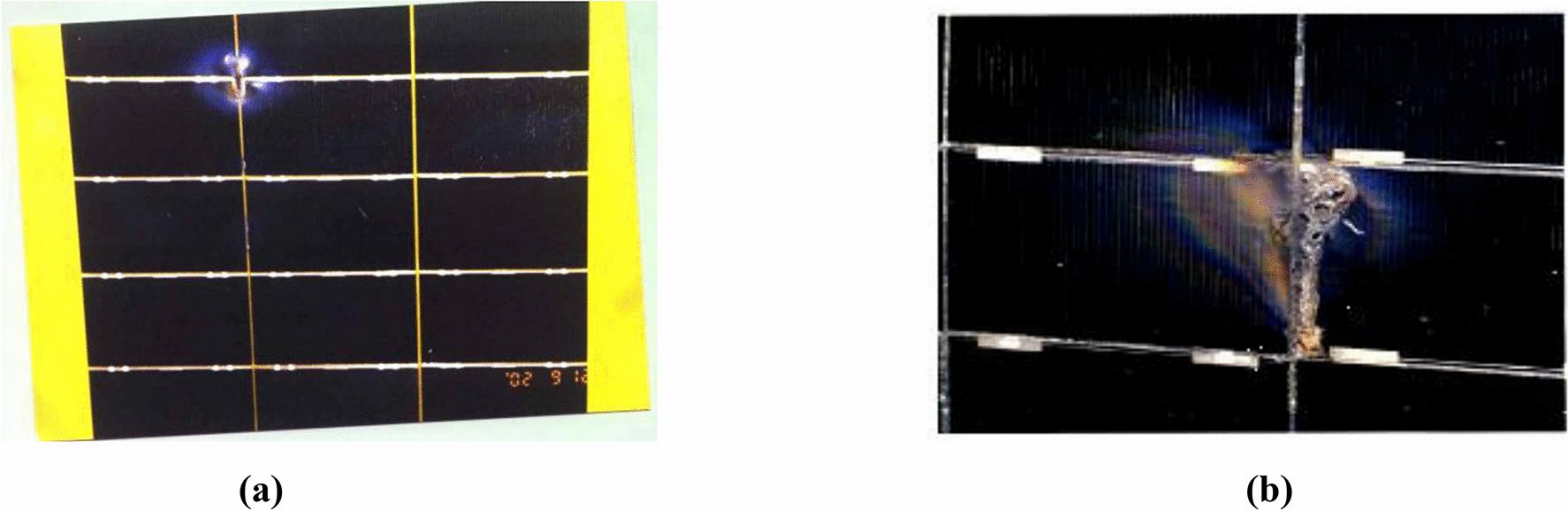
(**a**) Sustained arc and damaged part of the array interconnects, and (**b**) Failure caused by ground discharging and arcing.

Figure 1a shows an illustrative example of the induced sustained arc initiated through the adjacent cells exposed to plasma^15^. This can cause damage to the cells and substrate leading to considerable power losses^24^. At high voltage, such arcs occur at the cell or array edges. An intensive arc occurring in the middle of a string of cells at a bias voltage of 440 V is demonstrated in Fig. 1b. The figure reveals that the array sample failed during the ground test^30^.

Furthermore, in the work of Momoh and Button^31^, a new approach to detecting photovoltaic panel loss is established, and aerospace arcing faults are analyzed based on an artificial neural network (ANN) method. Alaa et al.^32^, studied the problem related to frequent arcs in photovoltaic systems using an ANN structure. Deep learning models are used to detect and classify series arc faults in photovoltaic panels.

The presented research work is a preliminary study on the behavior of discharging and arcs on solar cell and array surfaces using image processing and deep learning techniques. A deep learning model involving CNNs and transfer learning techniques has been applied to analyze images of arcs on solar cell surfaces in an attempt to understand the spatial variation in the arced and defective regions. The structure of the paper is as follows. “Proposed methodology” section describes the methodology used in this research, and explains the preprocessing and dataset. “Deep learning (DL) model and convolutional neural network (CNN)” section discusses the proposed model architecture and training followed by the applied techniques. The analyses of the applied methods and the obtained results are listed and discussed in “Results and discussion” section. Conclusions and recommendations for future work are presented in “Conclusions and future work” section.

## Proposed methodology

### Solar array and the tested cells

**Fig. 2:**
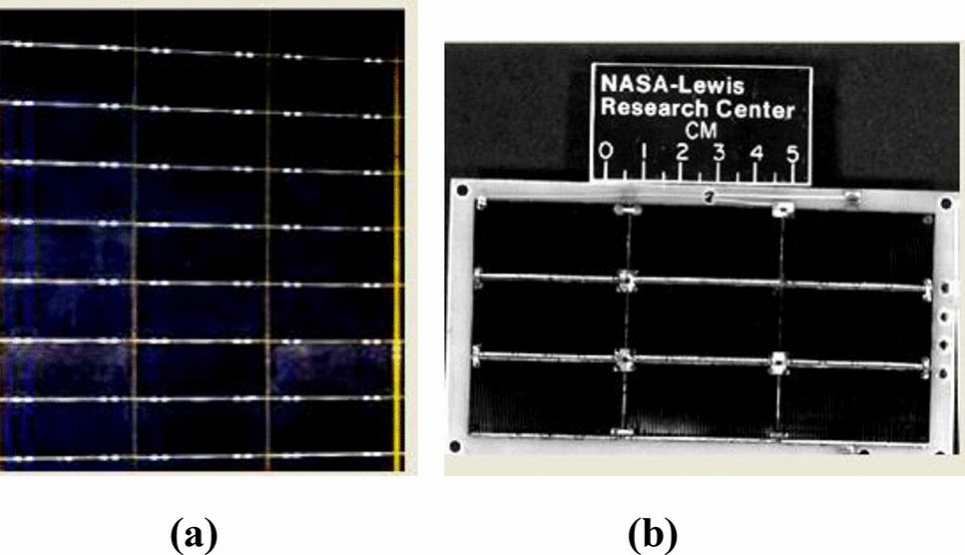
(**a**) Solar array sample, and (**b**) the size of the cells used in the test.

**Fig. 3:**
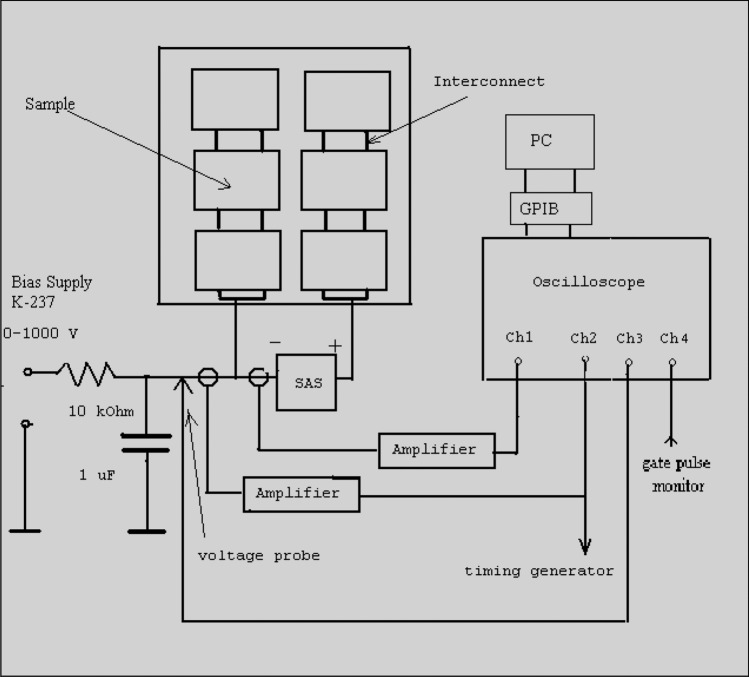
A schematic diagram of the circuit used for arc study.

Figure 2a shows pictures of a solar array sample of cells used in the experimental simulation. All dielectric–conductor junctions close to one interconnect area are insulated by tape to avoid spectral arcs between cells. The size of the sample cells is depicted in Fig. 2b^26^. Detailed description of ground based simulation tests are discussed and presented in the works of Refs.^15,33^. A circuit diagram for studying the arc behavior is shown in Fig. 3^15,34^.

**Fig. 4:**
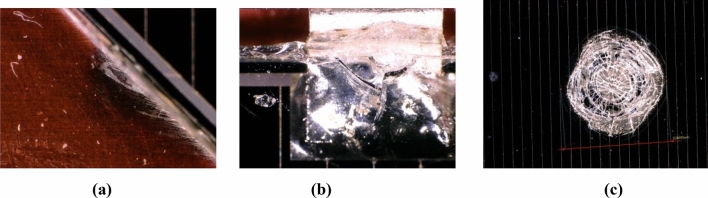
Images of (**a**) cell edge arc site, (**b**) interconnect contamination, and (**c**) mid -cell arc site.

Figure 4 illustrates the micrographic images of a sample of the cells in an array coupon shown in Fig. 3 after electrical discharges and arcs caused by the plasma impact. The arcing was recorded using a video camera at 30 frames per second to determine the arc site location. Arc sites are identified on interconnects and the cell edges^35^. The figure displays the arcs observed at different positions of the cell surface: the cell edge, the interconnector with contamination, and the mid-cell. This is shown as Fig. 4a–c respectively. The images were captured by Prof. Boris Vayner at the Air Force Research Laboratory (AFRL)^35^. The image in Fig. 4a shows arc discharge on the cell edge surface. These arcs can cause the ejection of metal atoms from the arc site resulting in contamination at the cell edges and spacecraft surfaces^20,29,35^. Contaminations observed at the interconnected points near and at the cell edges are shown in Fig. 4b. The contamination is caused by the inverse gradient discharges located on triple junctions^35^. For edge and contaminant surface, dimensions are about 5–8 mm. Figure 4c displays breakdown and micro-cracks formed on the cell coverglass. The scale shown for the breakdown area is 8495 µm (8.5 mm–1 cm). So, the number^1^ refers to the diameter of a spot shown in the picture—approximately 1 cm. The thickness of the coverglass plus adhesive is about 150 µm (0.15 mm). The coverglass breakdown occurred under normal gradient discharge.

The images shown in Figs. 4 were taken from a microscope in open air, consequently, the brightness is influenced by the reflection of light. The damaged areas observed in the images are not only caused by the arc itself but also by environmental conditions. That is why these images show more damaged and bright areas^35^. These images are analyzed to understand the behavior and spatial variation in arc discharges on the cell and array surfaces.

### Deep learning techniques

#### Dataset overview

In this section, it should be noted that, we could not obtain digital data or additional image data from the ground tests. Therefore, the dataset used for this study is limited. The dataset includes various types of solar cell defects, such as material degradation, micro cracks, arcing discharge, and shading issues^36^. Not all defects in the training and validation dataset are caused solely by arcing. However, the validation dataset used to assess the model’s performance is focused specifically on arc-induced defects.

The dataset provides annotations for each image, indicating the defect probability (ranging from 0 to 1) and the type of solar module (either monocrystalline or polycrystalline) from which the solar cell is extracted. All images have been normalized for size and perspective, and any lens-induced distortions are corrected prior to extraction, ensuring consistency and accuracy in the dataset. A comprehensive dataset specifically designed for benchmarking the visual identification of defective solar cells in electroluminescence (EL) imagery is utilized in this research. The dataset consists of 2624 high-resolution grayscale images (300 × 300 pixels) of solar cells extracted from 44 different photovoltaic modules. The images include both functional and defective cells with varying degrees of degradation, which can adversely affect the power efficiency of solar modules^36,37^.

#### Dataset usage

The dataset used in this study consists of high-resolution electromagnetic images of solar cells, categorized based on their condition. To facilitate its use, the dataset is organized into two main components:

1. *Images Directory* All images are stored in the “images” directory. Each image represents a solar cell in a specific state (functioning or defective) and is provided in a standardized format (300 × 300 pixels, 8-bit grayscale).
2. *Annotations File* The corresponding metadata and annotations for the images are stored in a CSV file named labels.csv. This file includes critical information such as:
  *Image Filename* The name of the image file.
  *Probability* The likelihood of defectiveness for each solar cell.
  *Type* The specific defect type or classification label.

To streamline the dataset’s usage in Python-based workflows, the elpv_reader utility is available in the referenced repository. The utility enables users to efficiently load and preprocess the dataset with minimal effort^36–38^. Below is an example of how to use this utility:

- from elpv_reader import load_dataset
- # Load images, defect probabilities, and types
- images, proba, types = load_dataset()

This code snippet automates the loading process and outputs three components:

- *images* A NumPy array containing the image data.
- *proba* A NumPy array of probabilities indicating defect likelihood.
- *types* A list of defect types or labels.

Requirements and licensing

- *Dependencies* The elpv_reader utility requires the installation of the following Python libraries:
  - NumPy
  - Pillow
- *License* The dataset is distributed under the Creative Commons Attribution-NonCommercial-ShareAlike 4.0 International License, which permits use for non-commercial purposes with proper attribution and requires sharing derivative works under the same license.

This structure ensures that the dataset is both accessible and user-friendly, facilitating its integration into various machine learning pipelines.

#### Data preprocessing and enhancement

The dataset is enhanced through preprocessing techniques, such as image contrast adjustment and feature highlighting, to improve the visibility of defect features and reduce misclassification risks associated with ambiguous labels. This ensures that the model is focused on visually evident defect patterns.

##### Image preprocessing and enhancement

Image preprocessing is a crucial step in any automated vision task. It involves preparing the raw image data in a way that enhances the performance of the model. We use a dataset of images of both healthy and defective solar panels to serve as the foundation for our model. Initial preprocessing involved resizing all images to a standard dimension of 224 × 224 pixels, followed by balancing the dataset using SMOTE (Synthetic Minority Oversampling Technique) to address class imbalances.

To improve the quality of the dataset and enhance defect visibility, we applied a series of preprocessing techniques before feeding the images into the model. These preprocessing steps are designed to ensure that defect features are more distinguishable, reducing the risk of misclassification due to ambiguous or low-contrast regions. The key preprocessing techniques used are as follows:

- *Contrast enhancement* Contrast limited adaptive histogram equalization (CLAHE) was employed to improve image contrast. CLAHE enhances local contrast, making subtle defect patterns more prominent while preventing over-amplification of noise. This technique ensures that critical defect details are more visible, aiding in better feature extraction during model training.
- *Feature highlighting (edge enhancement—initial testing phase)* During the preliminary analysis, Sobel edge detection filters were tested to emphasize vertical and diagonal edges, which could help in detecting defect patterns based on sharp intensity variations. However, after evaluating model performance, this step is not included in the final preprocessing pipeline as it does not significantly improve classification accuracy. The decision to omit this step is based on empirical results, which show that contrast enhancement alone is sufficient for highlighting defect features effectively. By implementing this preprocessing pipeline, we ensure that the model is focused on visually evident defect patterns, reducing the misclassification risks associated with ambiguous labels. The results demonstrate an improvement in model robustness by enhancing key defect features while maintaining the original structural details of the images.

##### Validation approach

The validation dataset is created to focus specifically on arc-induced defects, which are more visually distinct. While the broader dataset includes ambiguities in labeling, the validation process prioritized images with clear defect patterns to mitigate the influence of non-confident assessments.

It is crucial to carefully analyze as much data as possible, and we refine the dataset to ensure that the model can learn generalizable patterns.

##### Grayscale image equalization

This technique adjusts the contrast of images by spreading out the most frequent intensity values. By applying equalization, it assures that the images have a uniform distribution of intensity values, making it easier for the model to detect image defects.

##### Grid-based clutch creation

Using OpenCV, a grid with a size of (8 × 8 pixels) is applied to the images. This method helps in normalizing lighting conditions across the image, ensuring that different parts of the image are processed uniformly.

##### Handling class imbalance with oversampling

The dataset had an unequal number of images labeled as defective and non-defective, which could have led to a biased model. To address this, oversampling techniques are applied using sklearn’s oversampling functions. These generated additional samples for the minority class to balance the dataset and ensure that the model did not favor the majority class. To ensure that the data required for training are in optimal condition, preprocessing steps have been performed.

#### Libraries used

Several powerful libraries have been employed to streamline the process of building, training, and testing the model.

##### OpenCV (open source computer vision library)

OpenCV is an open-source computer vision and machine learning software library. It contains over 2500 optimized algorithms for a wide range of applications, including image processing and objects detection. In this study, OpenCV is used extensively for image preprocessing tasks such as grayscale equalization, grid-based processing, and also for visualization tasks concerning both two and three dimensional analysis.

##### NBLToolkits.nblot3d

This library is utilized to create three***-***dimensional (3D) visualizations. By using NBLToolkits.nblot3d, we are capable of generating 3D surface plots that provide a deeper understanding of the intensity distributions across the images. This helped in analyzing the patterns and structures that the model might be focusing on during its predictions.

##### Keras (with TensorFlow backend)

Keras is an open-source software library that provides a Python interface for artificial neural networks. Keras acts as an interface for the TensorFlow library. In this study, Keras is used to build and train the CNN model, leveraging TensorFlow’s powerful computation engine. The ability to easily define complex neural networks with minimal code makes Keras an ideal choice for deep learning mission.

##### Scikit-learn (sklearn)

Scikit-learn is a free software machine learning library for the Python programming language. It features various classification, regression, and clustering algorithms, including support vector machines, random forests, gradient boosting, and k-means. Scikit-learn (sklearn) is used to handle class imbalance through oversampling, a critical step in ensuring that the model learns to recognize both defective and non-defective cells equally well. Therefore, we applied an equalization function to each image to standardize the pixel intensity distribution. This step enhances the contrast in the images, making the defects more detectable by the model. OpenCV’s functionality is used to create a grid with a size of (8 × 8 pixels). This grid allowed us to apply local processing techniques that help in normalizing the lighting conditions across different parts of the image.

**Fig. 5:**
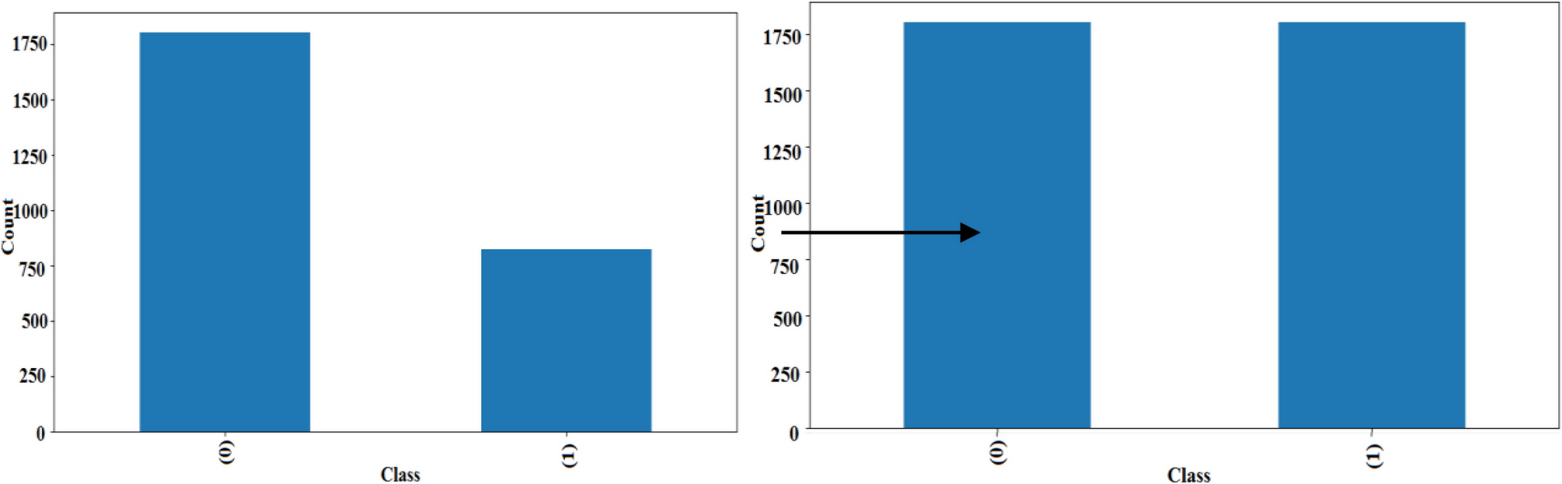
Data class distribution.

**Fig. 6:**
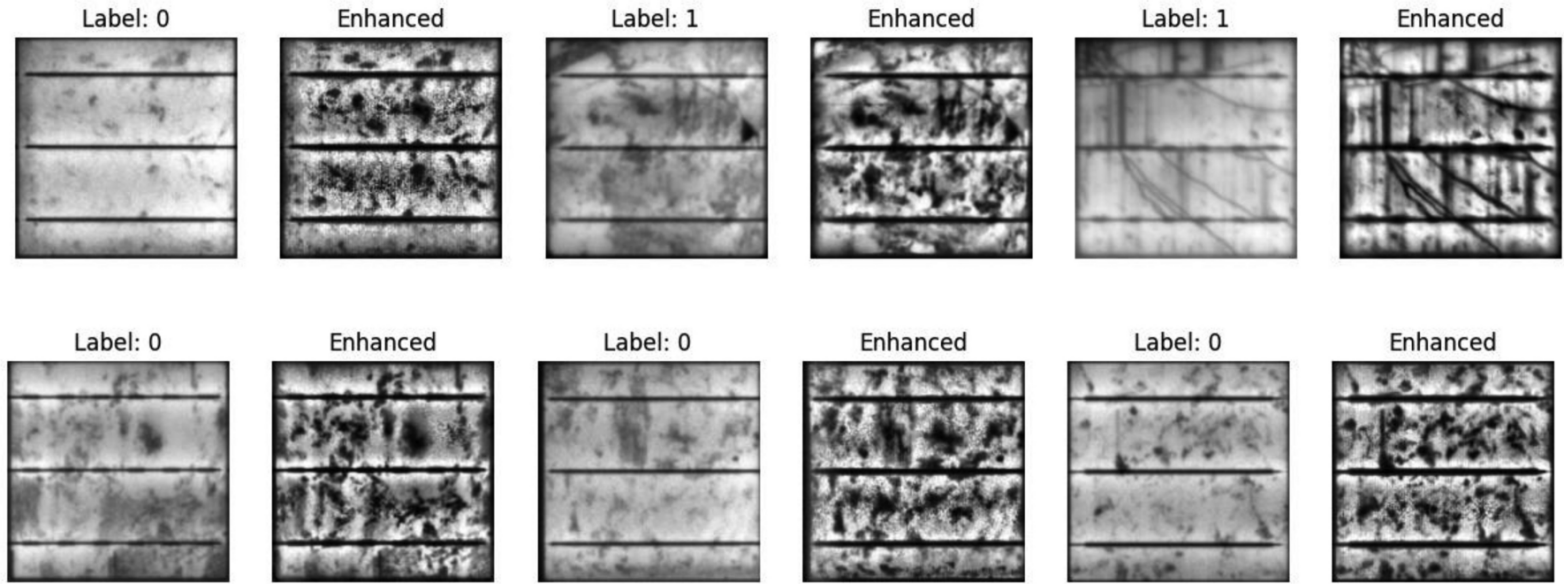
Samples of images and the improved version.

The dataset originally had an unequal distribution of the 0 and 1 labels, which could have biased the model (see Fig. 5). To address this, we applied oversampling technique using sklearn’s oversampling functionality. The left chart, in Fig. 5, represents the original dataset, which is unbalanced, meaning one class has significantly more samples than the other. This imbalance can negatively impact the model’s performance by biasing predictions toward the majority class. The right chart shows the dataset after applying a balancing technique, such as oversampling, undersampling, or synthetic data generation (e.g., SMOTE). Both classes have equal representation, which helps the model learn more effectively and make fairer predictions across all categories. The black arrow illustrates the transformation of the dataset before and after applying the balancing technique. This technique generates additional samples for the minority class, thereby balancing the dataset and improving the model’s ability to generalize. Examples of images with the enhanced versions are shown in Fig. 6.

## Deep learning (DL) model and convolutional neural network (CNN)

Deep learning is a subset of machine learning that deals with algorithms inspired by the structure and function of the brain called artificial neural networks. These algorithms learn from large amounts of data, making them especially powerful in tasks like image recognition, natural language processing, and more^39,40^. In this dedicated study, we focus on building a deep learning model to predict defective areas of solar cells. The dataset used consists of 2624 grayscale images divided into an 80–20% split between training and validation sets. This distribution ensures sufficient data for training while maintaining a robust validation process. Each grayscale images sized at 300 × 300 pixels and extracted from high-resolution electromagnetic sensor images of photovoltaic modules. These images are normalized for size and perspective, with all distortions due to the camera lens removed prior to solar extraction. Each image is annotated with a binary label (0 or 1) indicating the presence of a defect and is further categorized by the type of solar module. The defects in the annotated images vary in nature, either intrinsic or extrinsic and are known to cause a reduction in the power efficiency of solar modules.

### Model architecture and training

Convolutional neural networks (CNNs) have been used for analyzing the arc images of solar array surface. CNNs are particularly well-suited for image data due to their ability to automatically detect patterns like edges, textures, and more complex features. A Convolutional Neural Network (CNN) typically consists of multiple layers, each serving a specific function. The explanation of the CNN framework is depicted as follows.

#### Problem formulation

We aimed to analyze arc images on solar array surfaces using a CNN-based deep learning technique^40^. The proposed framework could be utilized for more potential applications including:

- The detection of arced regions and defective solar cells.
- The classification of different types of arc-induced damage.
- The prediction of regions prone to future arc events.

The input to the model is a set of grayscale images, each pre-processed to remove distortions and normalize the data. The output is a classification indicating whether a cell is defective or intact.

### CNN architecture

A typical CNN involves the following key components:

i. *Convolutional layer* The convolution operation extracts different features such as edges, shapes, and textures from the images. Each convolutional layer applies a set of learnable filters, which slide over the image and perform a dot product between the filter and small patches of the image.
  Mathematically, for an input image $X$ and a filter $F$, the output feature map $Y\left( {i,j} \right)\,$ is formulated as
  1 $$Y\left( {i,j} \right)\, = \sum\nolimits_{m} {\sum\nolimits_{n} X } \left( {i + m,j + n} \right) \cdot F\left( {m,n} \right),$$
  where the symbols $\,i,j$ denote the spatial coordinates, and $n,m$ iterate over the filter dimensions.
ii. *Activation function (ReLU)* The ReLU (Rectified Linear Unit) function introduces non-linearity into the network. This function is defined as:
  2 $${\text{Re}} LU\,\left( x \right) = \max \left( {o,x} \right).$$
  This ensures that the network can model complex, non-linear relationships.
iii. *Pooling layer* Pooling reduces the spatial dimensions of the feature maps, which helps in making the model computationally efficient and less prone to overfitting. Max pooling is commonly used, which selects the maximum value from a patch of the feature map. For example, max pooling over a 2 × 2 region is written as:
  3 $$Y\left( {i,j} \right)\, = \max \,\left[ {X\left( {i\,,\,\,j} \right)\,,\,X\,\left( {i + 1\,,\,\,j} \right)\,,\,X\left( {i\,,\,\,j + 1} \right)\,,\,X\left( {i + 1\,,\,\,j + 1} \right)} \right].$$
iv. *Fully connected layer* After several convolutional and pooling layers, the feature maps are flattened and passed through fully connected layers to perform the final classification and predictions. The output is a binary classification for detecting defects.

### CNN architecture proposed for image analysis

Training and validation accuracies have been tested with two different model architectures. The first model is a custom-built Convolutional Neural Network (CNN) with various layers. The CNN architecture is designed with several convolutional layers, followed by pooling and dense layers, making it adept at learning the intricate details required to distinguish between different parts of defective solar cell surfaces.

### Model design and development

This section presents a comparative analysis of two models for binary classification tasks. The first model is designed and trained from scratch, while the second employed transfer learning using a pre-trained EfficientNetV2L model. Both approaches are described in detail below, with their respective architectures, training procedures, and performance metrics compared comprehensively.

#### Model 1: custom CNN architecture

The first model is a custom Convolutional Neural Network (CNN) designed from scratch, optimized for image classification tasks. The architecture includes the following key components:

1. *Input layer* The input shape is defined as 128 × 128 × 1128 × 128 × 1128 × 128 × 1 for grayscale images.
2. *Convolutional layers* Two Conv2D layers with kernel sizes of 3 × 33 × 33 × 3 and 128,128,128 filters in the final layer, employing ReLU activation.
  Regularization using L2L2L2 norms is applied to reduce overfitting.
  Batch normalization is implemented after each convolution to stabilize training.
3. *Pooling layers* MaxPooling layers with a 2 × 22 × 22 × 2 kernel size to reduce spatial dimensions.
4. *Dropout layers* Dropout rates of 30% are applied after convolutional and dense layers to prevent overfitting.
5. *Dense layers* A fully connected dense layer with 512 neurons before the output layer.
6. *Output layer* A single neuron with a sigmoid activation function for binary classification.

The sequence of the custom CNN model is summarized in Table 1.

**Table 1:** Sequential of the custom CNN model.

Layer (type)	Output shape	Param #
conv2d (Conv2D)	(None, 224, 224, 64)	640
batch_normalization (batch normalization)	(None, 224, 224, 64)	256
activation (activation)	(None, 224, 224, 64)	0
max_pooling2d (max pooling 2D)	(None, 112, 112, 64)	0
dropout (dropout)	(None, 112, 112, 64)	0
conv2d_1 (Conv2D)	(None, 112, 112, 128)	73,856
batch_normalization_1 (batch normalization)	(None, 112, 112, 128)	512
activation_1 (activation)	(None, 112, 112, 128)	0
max_pooling2d_1 (MaxPooling2D)	(None, 56, 56, 128)	0
dropout_1 (dropout)	(None, 56, 56, 128)	0
flatten (flatten)	(None, 401,408)	0
dropout_2 (dropout)	(None, 401,408)	0
dense (dense)	(None, 512)	205,521,408
dense_1 (dense)	(None, 1)	513
Total params: 205,597,185 (784.29 MB) Trainable params: 205,596,801 (784.29 MB) Non-trainable params: 384 (1.50 KB)		

##### Training configuration

- Optimizer: Adam
- Loss Function: Binary Crossentropy
- Epochs: 40
- Data Augmentation: None explicitly stated.

##### Performance

- Training Accuracy: 95.98%
- Validation Accuracy: 83.24%
- Training Loss: 1.065
- Validation Loss: 2.228

#### Model 2: transfer learning with efficientNetV2L

The second model leverages transfer learning using the EfficientNetV2L architecture as a base model. The pre-trained weights on ImageNet are utilized to initialize the layers, and the model is fine-tuned for the task.

1. *Base Model* EfficientNetV2L without its top layers. The input shape is 128 × 128 × 3128 × 128 × 3128 × 128 × 3 (for RGB images).
  Pre-trained weights allow leveraging learned features for faster convergence.
2. Added Layers:
  A flatten layer to transform the base model’s output.
  A dense layer with 512 neurons and ReLU activation.
  A dropout layer with a rate of 50% for regularization.
  An output layer with a single neuron and sigmoid activation for binary classification.
3. *Trainable layers* The base model’s layers are frozen during training, while the newly added layers are trainable.

The sequence of the transfer learning model is given in Table 2.

**Table 2:** Sequential of the transfer learning model.

Layer (type)	Output shape	Param #
efficientnetv2-l (functional)	(None, 7, 7, 1280)	117,746,848
flatten_1 (flatten)	(None, 62,720)	0
dense_2 (dense)	(None, 512)	32,113,152
dropout_3 (dropout)	(None, 512)	0
dense_3 (dense)	(None, 1)	513
Total params: 149,860,513 (571.67 MB) Trainable params: 32,113,665 (122.50 MB) Non-trainable params: 117,746,848 (449.17 MB)		

##### Training configuration

- Optimizer: Adam with a learning rate of 0.001
- Loss Function: Binary Crossentropy
- Epochs: 54
- Data Augmentation: Not explicitly stated.

##### Performance

- Training Accuracy: 91.02%
- Validation Accuracy: 89.05%
- Training Loss: 0.197
- Validation Loss: 0.271

### Comparison of models performance

An example of the parameters obtained from the two models is provided in Table 3.

**Table 3:** Parameters resulting from the models.

Metric	Model 1 (custom CNN)	Model 2 (transfer learning)
Train accuracy (%)	95.98	91.02
Validation accuracy (%)	83.24	89.05
Train loss	1.065	0.197
Validation loss	2.228	0.271
Epochs	40	54

{

“Model 1”: {

“Train Accuracy”: 0.9597780704498291,

“Validation Accuracy”: 0.8324099779129028,

“Train Loss”: 1.0650568008422852,

“Validation Loss”: 2.2284746170043945

},

“Model 2”: {

“Train Accuracy”: 0.9101941585540771,

“Validation Accuracy”: 0.8905817270278931,

“Train Loss”: 0.1970704197883606,

“Validation Loss”: 0.2714773416519165

}

**Fig. 7:**
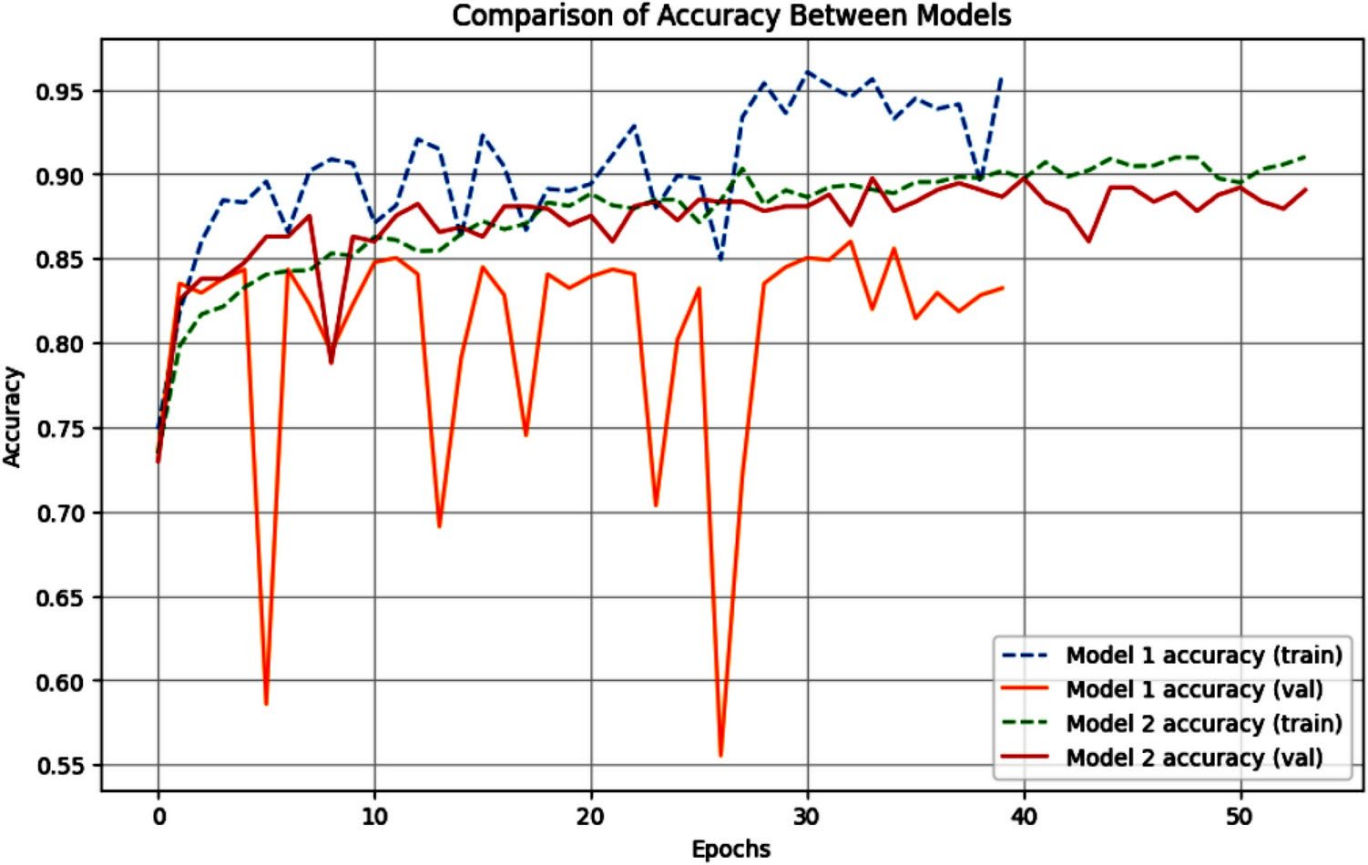
Comparison of the accuracy between the models.

**Fig. 8:**
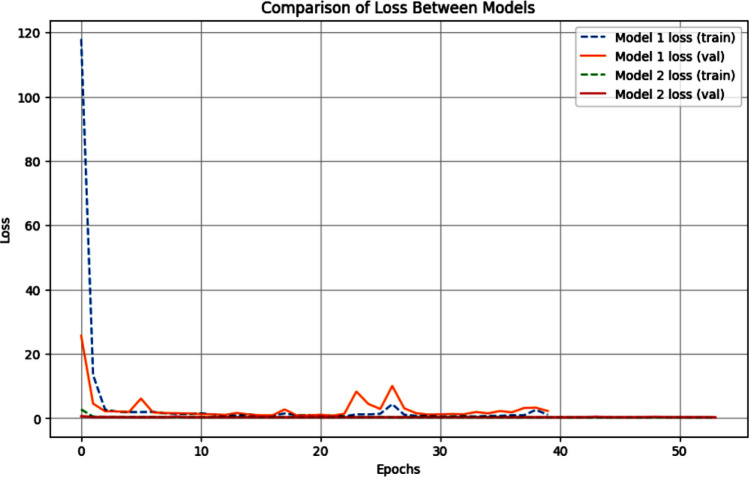
Comparison of loss between the models.

A comparison between the accuracy and loss of both models is shown in Figs. 7 and 8 respectively.

The obtained results have shown the following:

1. Accuracy:
  While Model 1 achieved higher training accuracy, its validation accuracy is significantly lower, suggesting possible overfitting.
  Model 2 exhibited higher validation accuracy, indicating better generalization.
2. Loss:
  Model 2 had substantially lower training and validation losses, demonstrating better convergence during training.
  Model 1’s high validation loss suggests difficulty in adapting to unseen data.
3. *Training time* Model 1 is trained in fewer epochs (40) compared to Model 2 (54). However, the additional epochs for Model 2 likely contributed to its superior generalization performance.

The results shown above present the trade-offs between custom CNN and transfer learning-based approaches. Model 1, while achieving high training accuracy, suffered from overfitting and reduced validation performance. In contrast, Model 2, leveraging the pre-trained EfficientNetV2L, demonstrated robust generalization capabilities with higher validation accuracy and lower loss values.

## Results and discussion

### Assessment and model testing

The model is rigorously checked on solar cell arc images. Most predictions are accurate, but the model failed to predict the image of the Fig. 4a correctly. This image regards as a challenge to the model, possibly due to subtle features or anomalies that confused the model. This may be attributed to the following reasons:

- The model’s misclassification, which may be due to a lack of exposure to diverse defective solar cell images during training, especially those captured under varying real-world conditions. The training dataset may have been biased towards ideal, lab-controlled images, limiting the model’s ability to generalize to more complex and less structured environments.
- Additionally, the image is taken at an oblique angle, while the training data predominantly featured images captured perpendicularly to the camera. This shift in perspective likely introduced variations in lighting and shadow effects, which the model, being unaccustomed to such conditions, struggled to interpret accurately.

Despite this, the overall performance is robust, with the model correctly identifying defects and faults in the remainder images used in the analysis.

### Three-dimensional (3D) image analysis

To gain deeper insights into the model’s predictions and understand the underlying patterns in the data, we conducted a 3D surface blur analysis using NBLToolkits.nblot3d and OpenCV.

It should be noted that, due to the limited number of available images, we employed a pixel intensity variation analysis to quantify differences between the affected and unaffected regions of the surface. However, our approach is based on relative surface curvature and intensity variations, which serve as indirect indicators of structural degradation. This approach allows us to establish a relative damage assessment metric despite the inherent challenges posed by illumination inconsistencies.

A surface blur analysis is a technique that allowed us to visualize the grayscale intensity values of the blurred images across the spatial dimensions. The model is applied to the images shown in the Fig. 4 from the dataset labeled as “Mid Cell Arc Site 2.jpg,” “Interconnect Arc Contamination 2.jpg,” and “Edge Arc Detail.jpg,” which are associated with defects and damaging to the cover glass. The preprocessing steps included resizing each image to 224 × 224,224 × 224,224 × 224, normalizing pixel values, and expanding their dimensions to match the model input requirements. The X-axis represents the horizontal position (width) of the image pixels, the Y-axis represents the vertical position (height) of the image pixels, and the Z-axis represents the depth intensity and magnitude of the arc events, with peaks representing the depth and arced intensity of the array surface. This indicates brighter regions and valleys indicating darker regions in the blurred image.

**Fig. 9:**
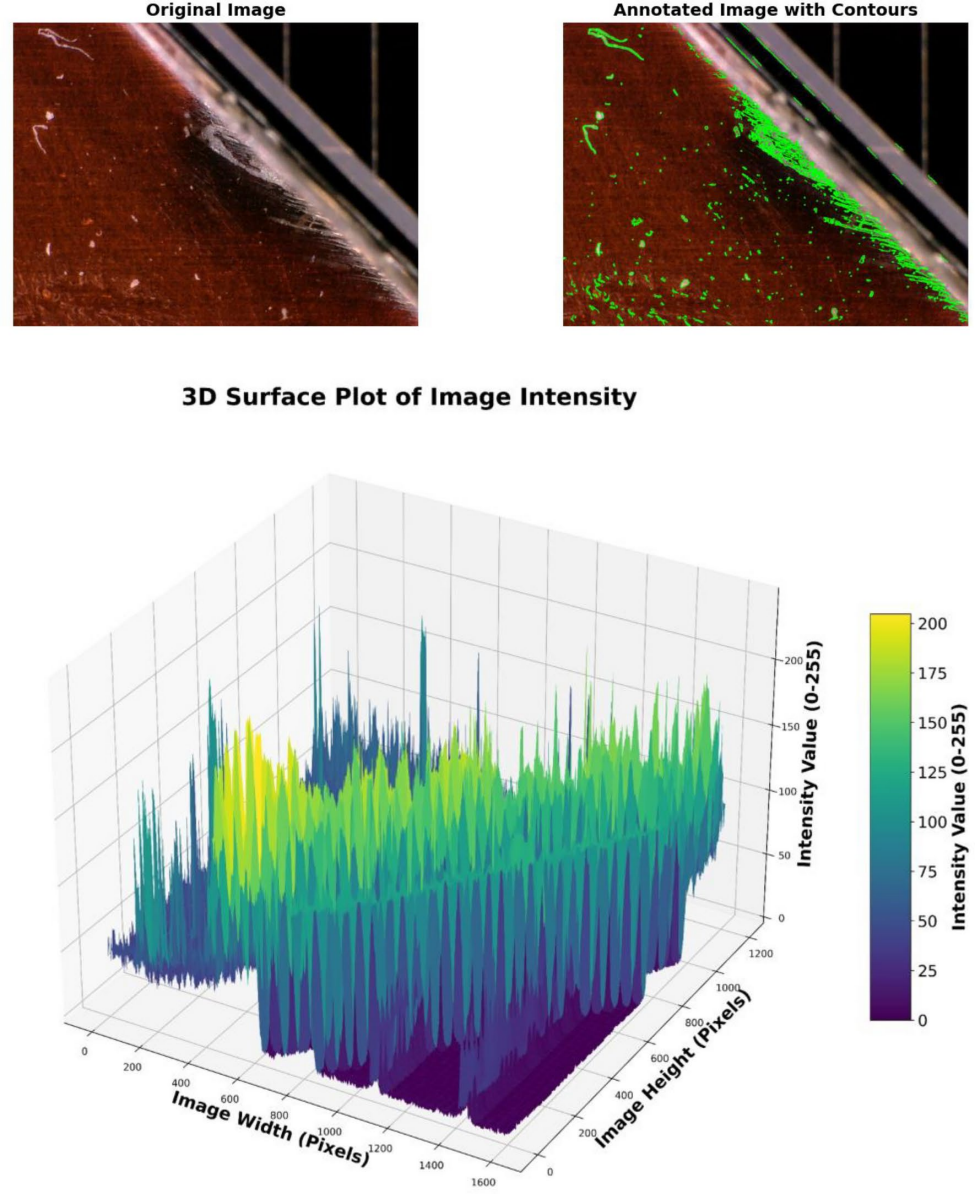
Three dimensional surface analysis of the defected area for cell edge.

**Fig. 10:**
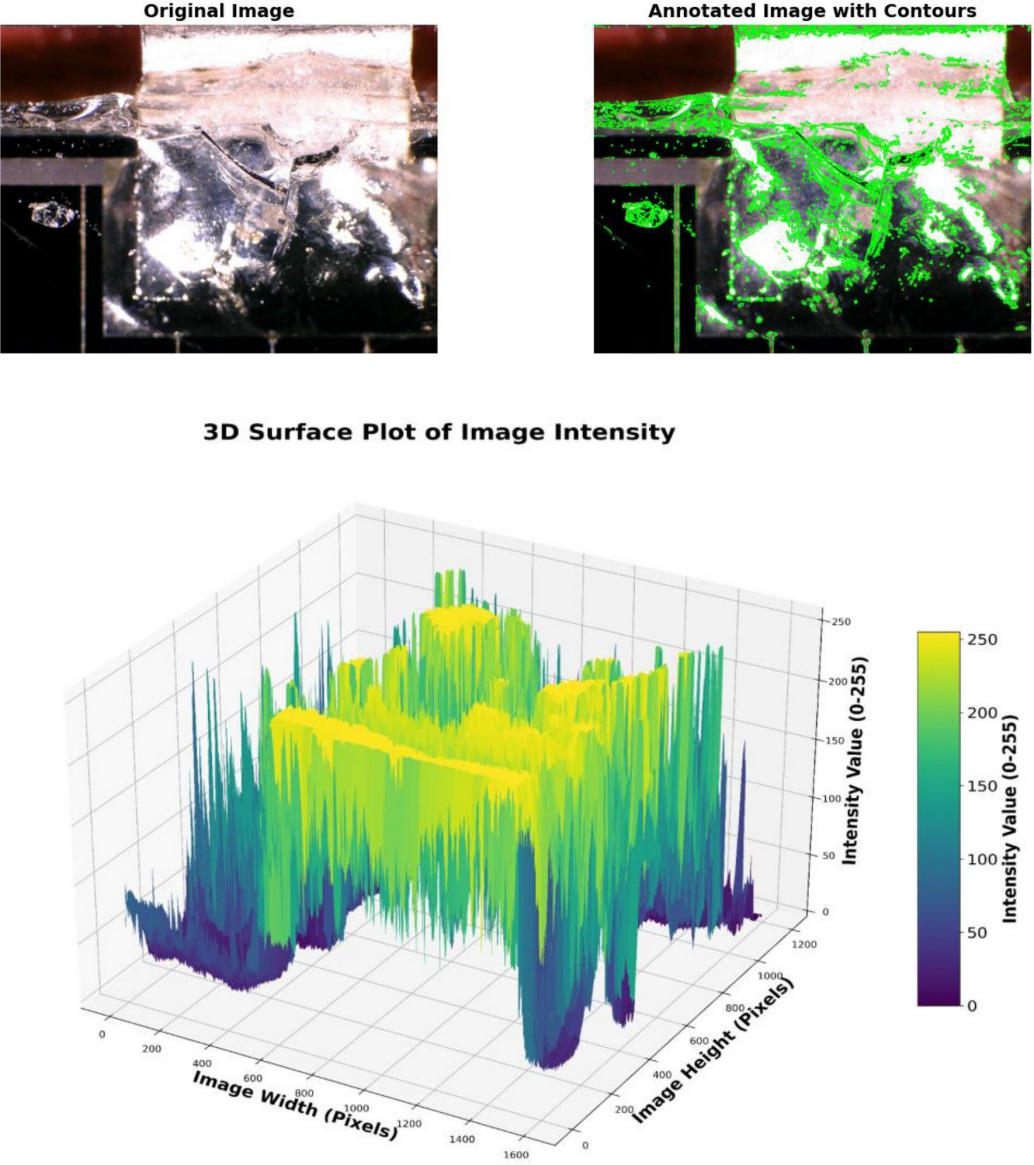
Three dimensional surface analysis of the defected area of interconnector.

**Fig. 11:**
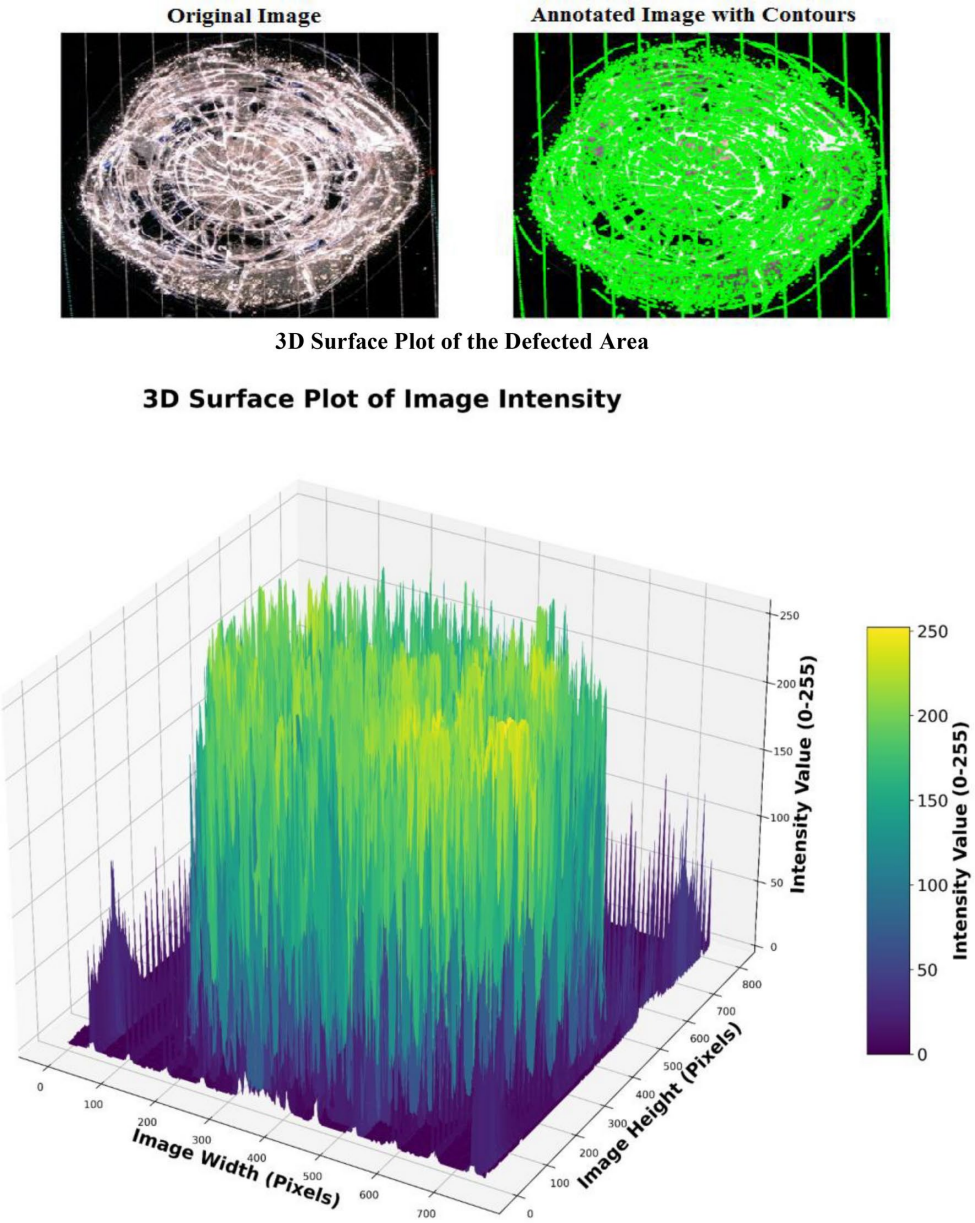
Three dimensional surface analysis of the defected area of the mid–cell.

Figures 9, 10, and 11, show the three-dimensional (3D) analysis and spatial distribution of the reflection intensities on the surface samples depicted in the images. The figures illustrate a 3D surface plot that visualizes the intensity values of a grayscale image. The X and Y axes correspond to the pixel coordinates of the image, while the Z-axis represents the intensity values, which range from 0 (black) to 255 (white). These plots are exploratory conducted to understand the depth intensity patterns. Thus, the third dimension represents the degradation and damaging appeared as the brightness of the cell surface. The axes also provide a sense of orientation, especially in 3D plots, where perspective is important to understand the data’s depth and their variation. The 3D graphs correspond to intensity mappings over the entire image area. A colormap is applied to highlight variations in pixel intensity, with purple representing lower intensity values and yellow indicating higher intensity values. This helps in analyzing the distribution of pixel intensities, detecting edges, and identifying patterns in the image. In the figures, image processing techniques are applied for analyzing the structural patterns in a crushed and degraded surface. The left image, labeled “Original Image,” presents the unprocessed input, showcasing a circular glass fracture pattern, while, the right image, labeled “Annotated Image with Contours,” displays the result of applying a contour detection algorithm to highlight the structural details of the degraded and damaged areas. Contours have been overlaid in green, capturing the intricate cracks and fragment boundaries. The contours are extracted using edge detection and thresholding techniques, which enhance the visibility of fine details. The identified contours help quantify fracture propagation patterns, offering insights into crack formation and structural integrity.

The analyses have shown that areas with sharp intensity changes in the original image manifest as more abrupt changes in the Z-axis value. The surface appeared smooth due to the gradual blur, which softened the transition between different intensity values.

The results have shown that the model’s prediction for the uploaded images indicated the following results:

- Image 1 (“Mid Cell Arc Site 2.jpg”) Prediction: The solar cell is defective.
- Image 2 (“Interconnect Arc Contamination 2.jpg”) Prediction: The solar cell is defective.
- Image 3 (“Edge Arc Detail.jpg”) Prediction: The solar cell is defective.

This reveals the model’s ability to successfully identify defects linked to arc-induced processes across multiple images. These predictions align with the defect features described in Ref.^37^, where the defects and associated damage present distinguishable patterns and thermal signatures. The preprocessing and prediction workflow ensure reliable detection, showcasing the model’s ability to extract and distinguish arc-related defect patterns effectively from the dataset.

### Two-dimensional (2D) images analysis

**Fig. 12:**
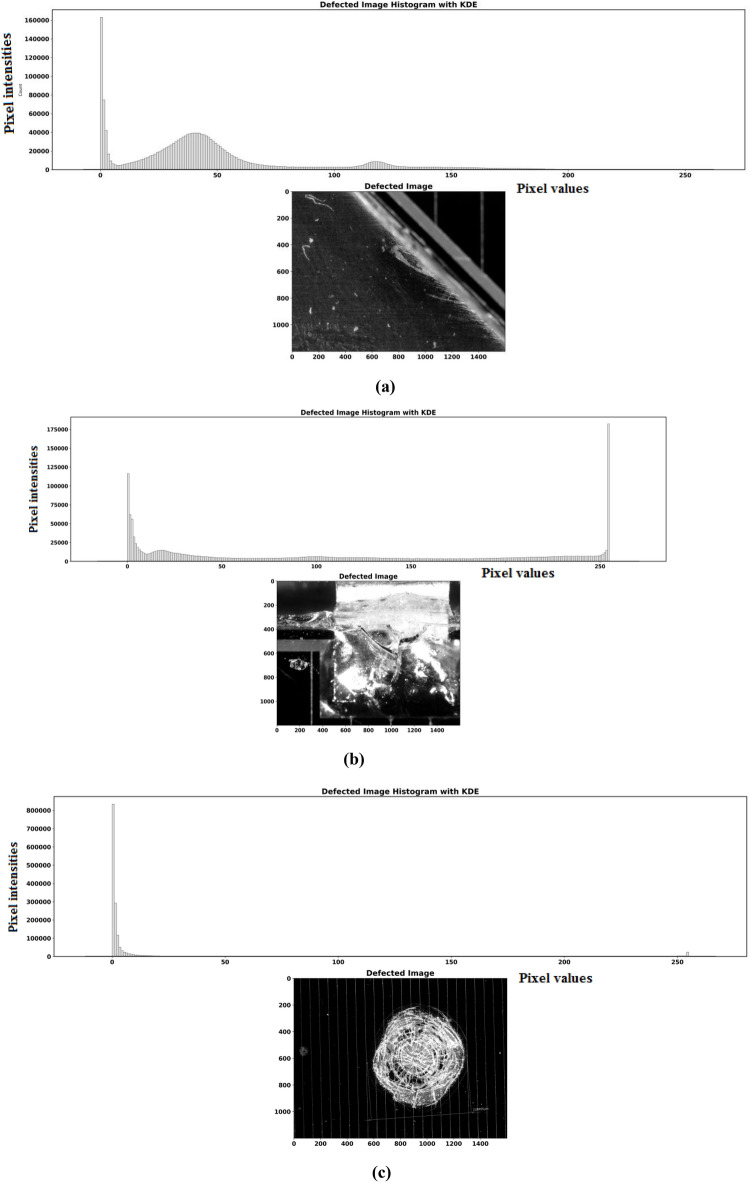
2D analysis of strength and arc intensities distributed on the surface area: Defected image histograms with KDE method.

A two-dimensional (2D) analysis is performed to understand the distribution of the reflection intensity of the damaged and arced regions. The histograms display the positions of the deformation and variations in brightness for the images. We compute pixel intensity histograms and contrast variations across the surface area to ensure that the observed differences between the affected and unaffected regions of the surface are not solely attributable to lighting artifacts but rather to underlying structural changes. CalcHist from OpenCV is used to estimate the histogram of the pixel intensities and evaluate the depth of the damaged region. This is plotted and shown in Fig. 12a–c respectively.

To enhance the visibility of more intense damaged regions in the grayscale image, the histogram of pixel intensities is computed using OpenCV’s cv2.calcHist function, which counts the occurrences of each intensity level (from 0 to 255). The histogram is flattened to a one-dimensional array to facilitate manipulation and visualization. A two-part plot is generated. The first part displays the scaled histogram using the matplotlib.pyplot.bar function with each bar representing the intensity value count after scaling. The second part shows the grayscale image, allowing direct visual correlation between the image and its pixel intensity distribution. To estimate the probability density function (PDF) of a continuous random variable, the Kernel Density Estimation (KDE) method is utilized. It is a non-parametric method used to estimate the probability density function (PDF) of a continuous random variable. Unlike histograms, which distinct the data into bins, KDE provides a smooth estimate of the underlying distribution by placing a continuous kernel (often a Gaussian function) at each data point. This smoothness helps in visualizing the distribution more effectively. In the image analysis or defect detection, KDE can be used to provide a smooth representation of pixel intensity distributions. This is especially useful for comparing the overall spread of pixel intensities between normal and defected images, as KDE offers a more flexible view compared to histograms, which may suffer from binning artifacts.

The results of the analysis have shown that the histograms reveal degradation and damaged areas of high and low reflection intensities within the defected regions of the images. For the surface, the areas of high reflection intensity and more brightness correspond to the defected and faulty region, while areas with lower intensity and less brightness are referred to as regions with fewer or no defects. This confirms that the model successfully performed a two-dimensional analysis of the images, providing further validation of its predictive capabilities. The analysis shows a consistent intensity difference (ΔI) between damaged and undamaged regions across multiple test cases, reinforcing the robustness of this method.

### Quantitative assessment of reflection intensity patterns

**Fig. 13:**
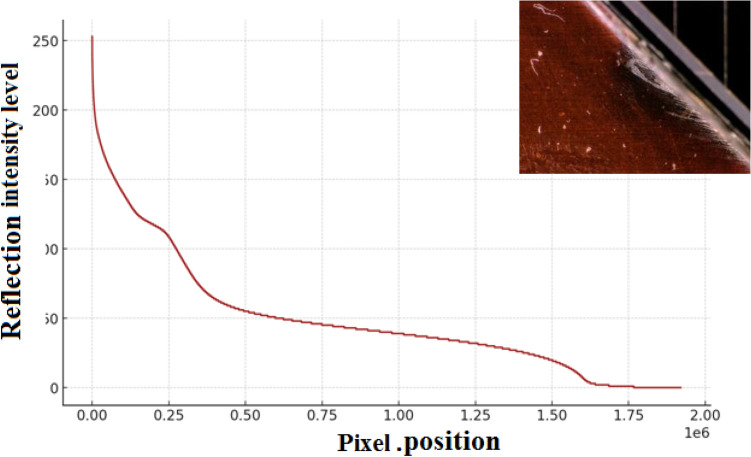
The reflection intensity distribution at the cell edge.

**Fig. 14:**
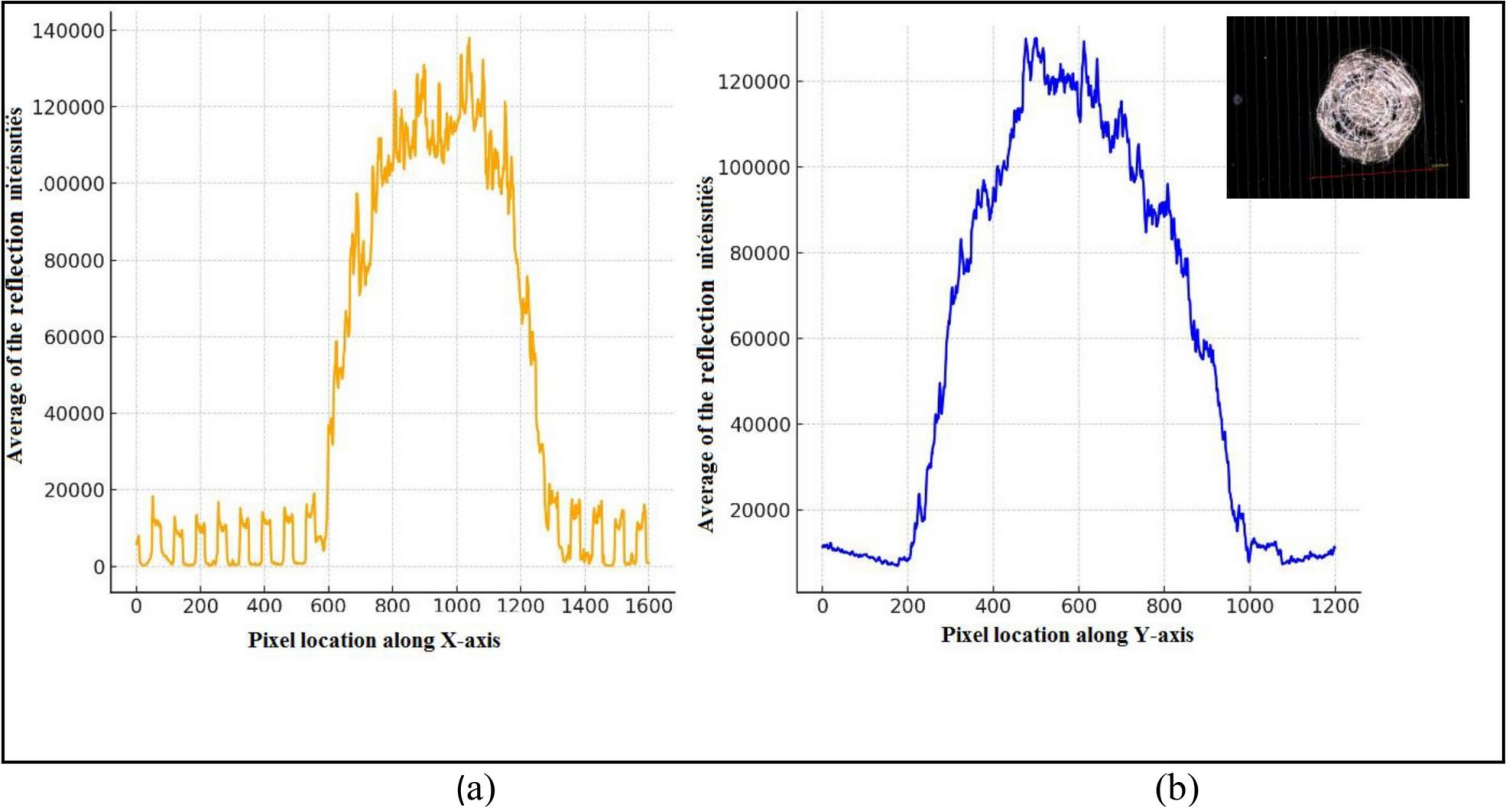
The variation in brightness and the reflection intensity distribution along the (**a**) X-axis and (**b**) Y-axis for the mid-cell.

Furthermore, the variation in the reflection intensities and brightness on the cell surface has been tested using Python code. This technique is applied to the images shown in Fig. 4a,c. The reflection intensity distribution for the images are then analyzed and shown in the Figs. 13 and 14, respectively.

To validate the variation in the arc behavior, Fig. 13 illustrates the analysis of the cell edge resulting from the application of Python. The graph shows the spatial distribution of the reflection intensity level for the image in Fig. 4a along the X-axis, arranged from the highest values to the lowest. The peak of the intensity value tends towards the maximum and gradually decreases, with a significant portion of pixels around a mid-range value. Finally, it tapers off towards zero. This reveals that the image contains a mixture of both high and moderately damaged areas around the cell edge region. Figure 13 provides additional insights into the distribution and variation of intensity values across the image, complementing the analysis in Fig. 12a.

In Fig. 14, the plots show the spatial distribution of the reflection intensity, for the image illustrated in Fig. 4c across both the X and Y axes of the mid-cell surface. The figure indicates that the reflection intensity values are at a maximum with more brightness and intense cracks around the center of the coverglass. A gradual decrease in the intensity level is observed outward, with noticeable deformation and irregularities in the intensity distribution along the path.

### Image analysis based on Maxim-DL techniques

To confirm the variation in brightness and reflection intensity distribution across the cell surface, Maxim-DL software is used to analyze the degraded and damaged regions of the surface in the same images 4a and 4c. The analysis is conducted based on the variation in brightness along the X-axis.

#### Intensity distribution of the arced region

**Fig. 15:**
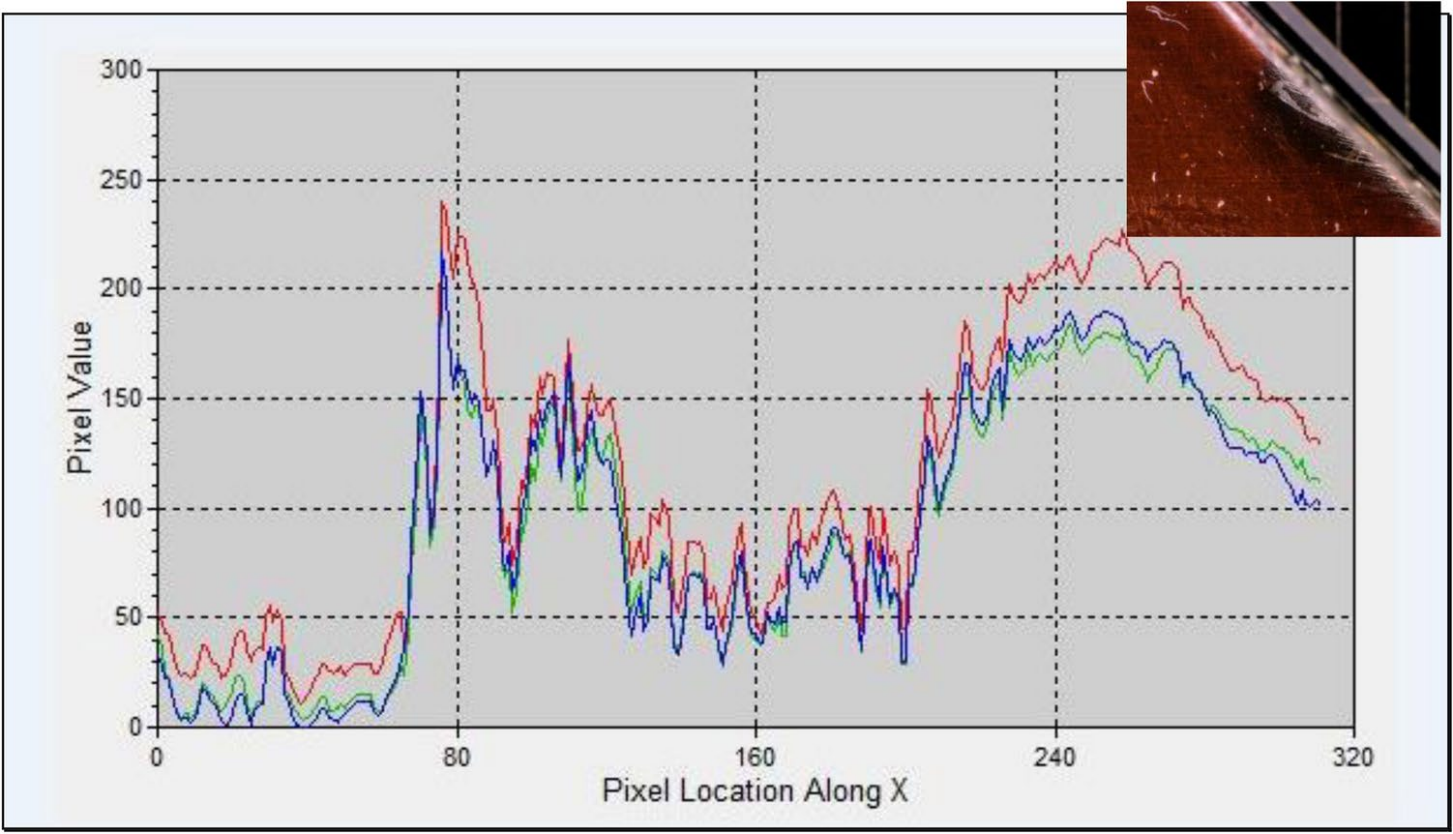
The reflection intensity distribution along the X axis for the cell-edge.

Figure 15 shows the reflection intensity distribution along the X-axis for the cell edge shown in Fig. 4a. The figure demonstrates that, the reflection intensity of the damaged and arced region is classified into three colors Red, Green, and Blue (RGB). These colors identify the variation in the reflection intensity from the highest value of brightness on the surface (Red color) to the lower one with a faint region (Blue color). Fluctuations and changes in the reflection intensity levels are noticeable with peaks and maximum values of both more and less intensive (RGB) regions along the cell edge.

**Fig. 16:**
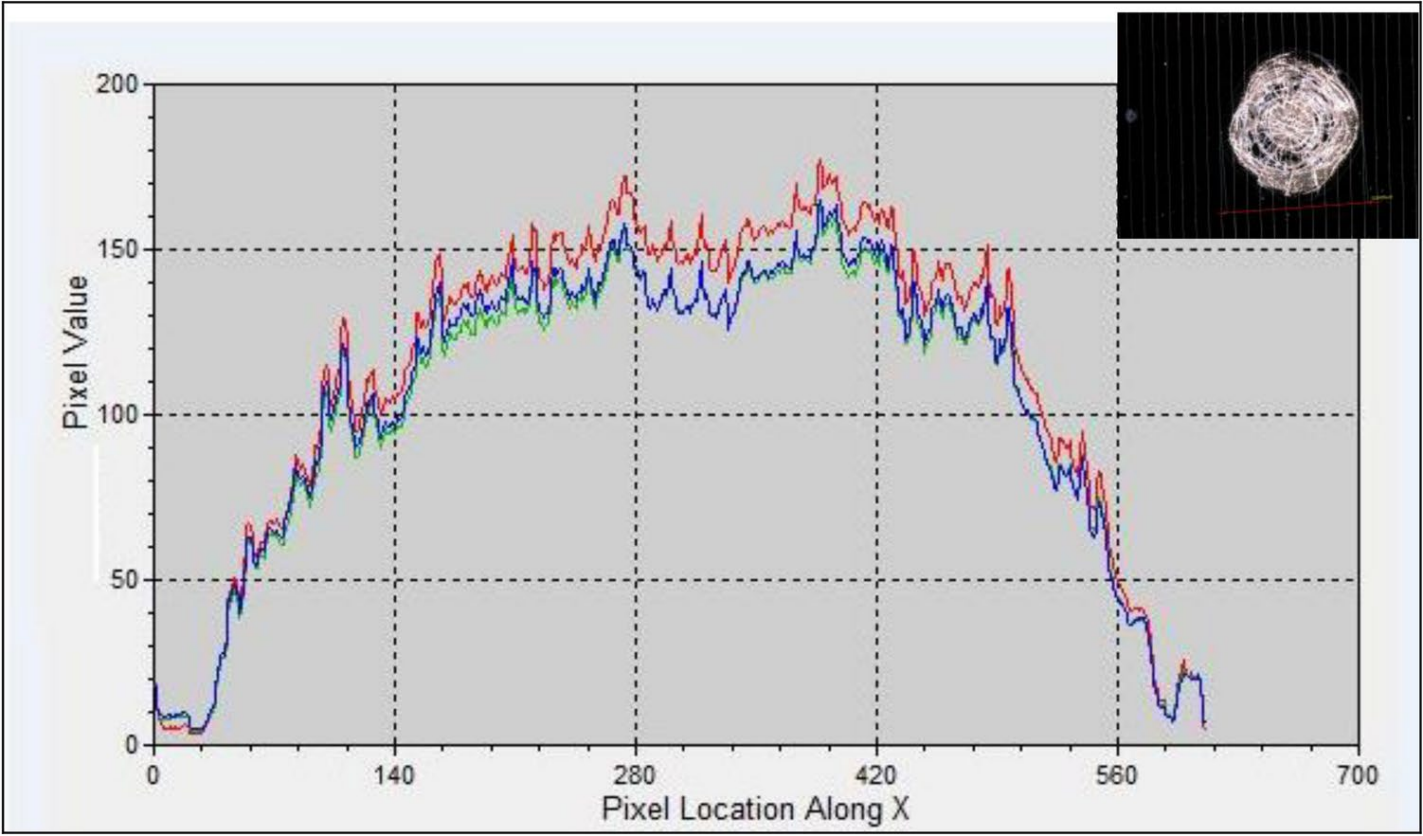
The reflection intensity distribution along the X axis for the Mid-cell.

The same relation is illustrated in Fig. 16 for the Mid-cell coverglass. The figure shows a different behavior, in which an intensive reflection is noticed with maximum values near the center of the arced region. These values gradually decrease outward.

#### Reflection intensity distributions across the cell surface

**Fig. 17:**
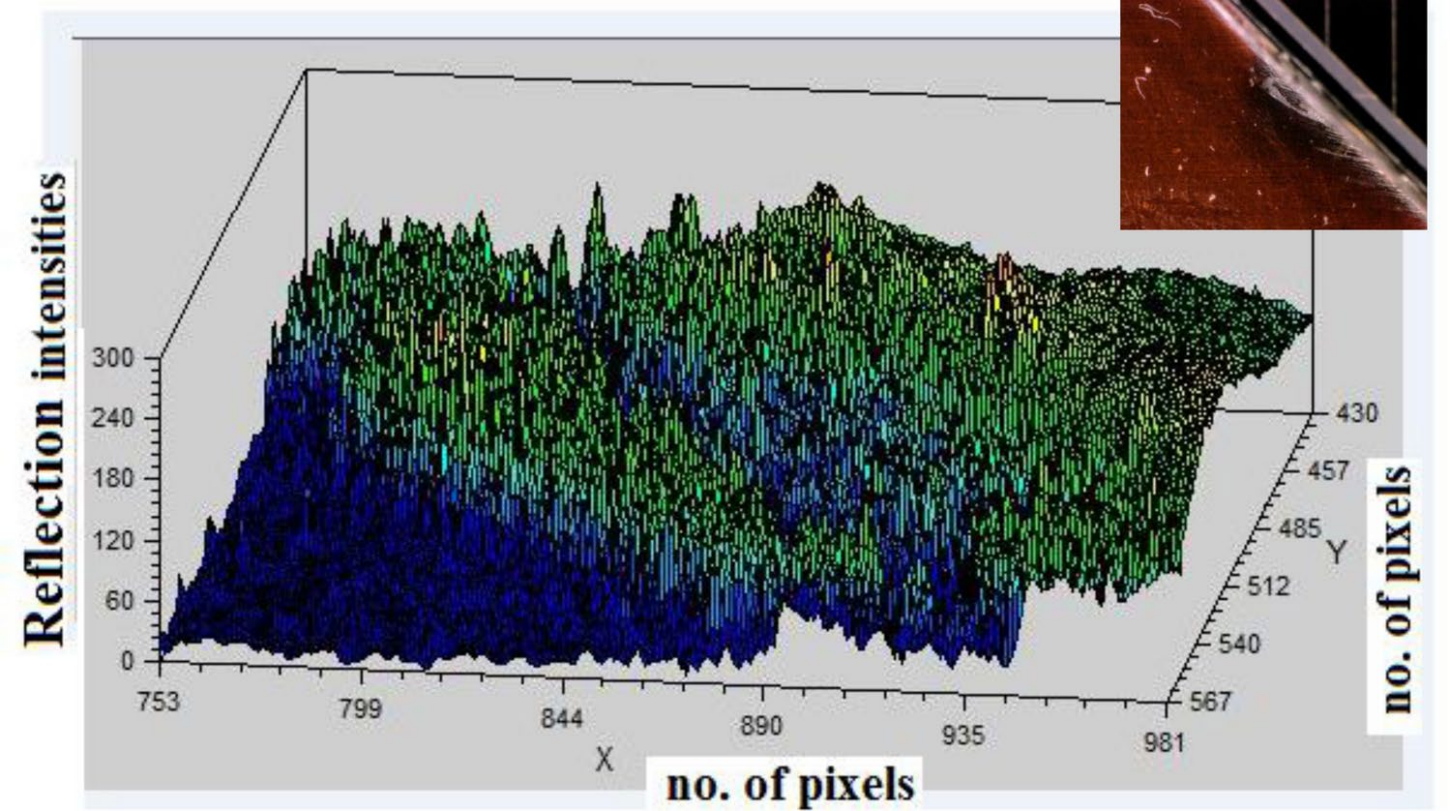
Reflection intensity distribution along the arced cell edge.

**Fig. 18:**
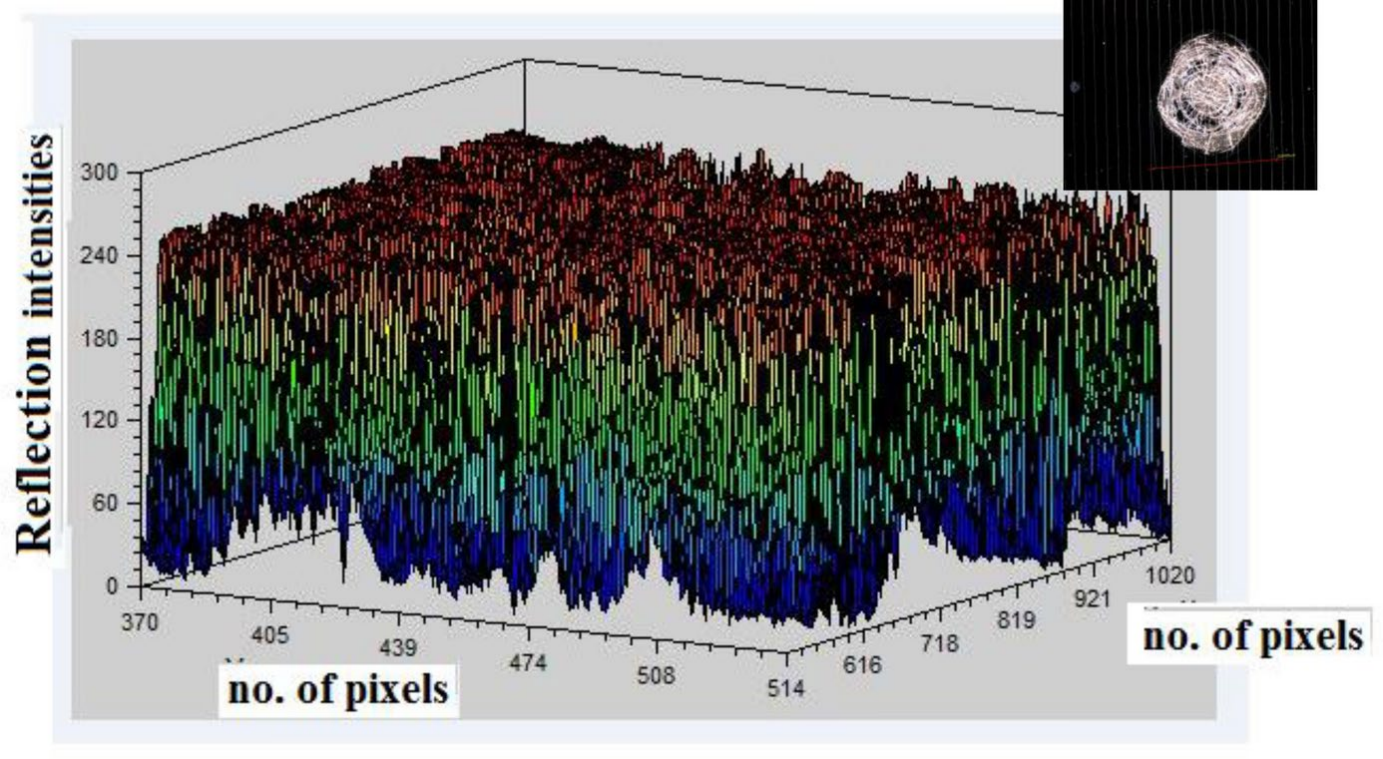
Reflection intensity distribution along the arced Mid-cell.

Figure 17 shows the reflection intensities distributed along the arced region of the cell edge. The figure describes the spatial distribution of the defected and damaged surface area. The analysis confirms the same behavior obtained for the variation in the reflection intensity levels with intensive regions along the cell edge. Moreover, the reflection intensity distribution along the damaged area of the Mid-cell is shown in Fig. 18. In this case, the three colors represent the arced region with the intensive and maximum reflection values to the lower ones. More brightness and reflection intensity levels are noticed with the maximum values (Red color) along the damaged area of the coverglass. This behavior is similar to that obtained from the 3D analysis plotted above for the mid cell coverglass surface. The results are comparable to those obtained applying the (DL) and (CNN) techniques shown in Figs. 9 and 11.

## Conclusions and future work

This research focuses on investigating the behavior of discharging and arcs on solar array surfaces due to plasma impact. Arc images are analyzed using image processing techniques, and artificial intelligence-based methods, particularly deep learning (DL) and convolutional neural networks (CNNs). Python code and Maxim-DL techniques are proposed to identify arc inception, reflection intensity, and variation in brightness of the arced regions. The study highlights the effectiveness of deep learning, especially CNNs and transfer learning, in identifying defective solar cells and array arcing based on arc images. Both 2D and 3D image analysis provide additional insights, improving the model’s interpretability and accuracy. The analysis of arc images confirms the more damaged regions observed at the mid-cells and interconnects. The results demonstrate that applying deep learning techniques is a promising approach for predicting and understanding the arc discharge. The combination of efficient data preprocessing, balanced class distribution, and advanced model architecture results in a robust and reliable model, suitable for practical applications in solar energy systems. The approach could lead to new techniques for studying the behavior and progression of damaging arc processes in solar arrays. Moreover, Python code and Maxim-DL qualitatively align with those derived from the deep learning technique regarding defective images and arc events.

This work provides valuable insights into image processing and analysis, offering recommendations for further applications of artificial intelligence (AI) in engineering and aerospace industries. The research may enhance the understanding of arcing processes and improve the predictive capabilities of AI models, supporting the design of more robust solar array systems for space applications. Future research will focus on simulations aimed at predicting the behavior of arc events using machine learning techniques applied to various scenarios involving arc currents, potentials and flashovers on solar arrays.

## Data Availability

The pictures shown in Fig. 4 and used in the analyses are provided from Prof. Boris Vayner himself^35^. The datasets generated during the current study are available from the work of the Refs.^36–38^. These datasets are licensed under a Creative Commons Attribution-NonCommercial-ShareAlike 4.0 International License.
